# Increased risk for developing gambling disorder under the treatment with pramipexole, ropinirole, and aripiprazole: A nationwide register study in Sweden

**DOI:** 10.1371/journal.pone.0252516

**Published:** 2021-06-01

**Authors:** Mirjam Wolfschlag, Anders Håkansson

**Affiliations:** 1 Department of Clinical Sciences Lund, Psychiatry, Faculty of Medicine, Lund University, Lund, Sweden; 2 Malmö Addiction Center, Clinical Research Unit, Malmö, Sweden; Chiba Daigaku, JAPAN

## Abstract

Gambling Disorder (GD) has recently been reclassified from an impulse-control disorder to a behavioural addiction and, as in other addictive disorders, the dopaminergic reward system is involved. According to neuroimaging studies, alterations within the striatal dopaminergic signalling can occur in GD. However, the findings to date are controversial and there has been no agreement yet on how the reward system is affected on a molecular basis. Within the last 20 years, there has been growing evidence for a higher risk to develop GD in response to certain dopaminergic medication. Especially the dopamine agonists pramipexole and ropinirole, and the dopamine modulator aripiprazole seem to increase the likelihood for GD. The goal of this study was to examine the association between a prescription for either of the three pharmaceuticals and a GD diagnosis in a large cross-sectional study of the Swedish population. Compared to patients with any other dopaminergic drug prescription (38.7% with GD), the diagnosis was more common in patients with a dopamine agonist prescription (69.8% with GD), resulting in an odds ratio of 3.2. A similar association was found between aripiprazole prescriptions and GD diagnoses, which were analysed within the subgroup of all patients with schizophrenia or a schizotypal, delusional, or another non-mood psychotic disorder. An aripiprazole prescription increased the likelihood of GD (88.8%) in comparison to patients without an aripiprazole prescription (71.2%) with an odds ratio of 3.4. This study contributes to the increasingly reliable evidence for an association between several dopaminergic drugs and a higher risk for developing GD. Therefore, one future research goal should be a better understanding of the neurobiology in GD to be able to design more selective dopaminergic medication with less severe side effects. Additionally, this knowledge could enable the development of pharmacotherapy in GD and other addictive disorders.

## Introduction

### Gambling disorder and the dopaminergic reward system

Gambling disorder (GD) is the permanent and severe manifestation of the gambling activity that many in the general population pursue sporadically as a hobby. Common forms are casino games, slot-machines, and lotteries, but in the last years internet gambling has grown in popularity [1]. The prevalence of GD worldwide ranges between 0.12–5.80% and under the special circumstances of the recent COVID-19 pandemic, GD has become an increasing problem for vulnerable individuals [2, 3]. Possible negative consequences of GD include a detrimental impact on close relationships, and higher mortality and suicidality [4–7]. Furthermore, GD is associated with different psychiatric comorbidities such as substance-use, depression, and anxiety [8].

Formerly seen as an impulse-control disorder, GD has been reclassified as a behavioural addiction and been renamed from “Pathological Gambling” in the latest revisions of the International Classification of Diseases (ICD 11) and the Diagnostic and Statistical Manual of Mental Disorders (DSM-5) [9, 10]. This decision was based on broad evidence for GD sharing many characteristics with substance-use disorders rather than impulse-control disorders, clinically and on a molecular basis [11–13]. Regarding the pathophysiology in GD, many different transmitter and hormone systems have been shown to be involved, including dopamine, opioids, serotonin, cortisol, and adrenaline [14]. Nevertheless, no clear understanding of the neurochemistry in GD could be obtained yet and there is no pharmacotherapy available specific for GD. As in other addictive disorders, opioid antagonists such as naltrexone have shown some beneficial effect but are insufficient as a sole approach.

One central element in the neurobiology of GD is the dopamine-based reward and reinforcement system [14]. Its main part are neurons within the mesolimbic dopamine pathway, that project from the ventral tegmental area in the brain stem into the nucleus accumbens (NAc) in the ventral striatum, located in the basal ganglia. Physiologically, this system enables the judgement of external stimuli, creates an evaluated memory for them, links them to each other, and conducts adequate behaviour [15]. In addiction, a certain substance or behaviour possibly over-activates the route of being given a positive value, even if the outcome is detrimental, for instance negative health effects. This leads to an impaired balance in the rating of stimuli and the subject of addiction receives pathologically increased attention, while other goals lose importance [16]. One important part in reward-related learning is Pavlovian conditioning, by which a neutral cue is linked to a secondary stimulus with a positive or a negative value [17]. The former unrelated cue becomes a conditioned stimulus which can from now on predict the secondary, unconditioned stimulus. On a molecular basis, this linkage is communicated by a shift from the basal tonic dopamine signalling at the NAc to phasic spikes. Whenever a hitherto neutral stimulus yields a more positive effect than expected, phasic signalling is the reaction to this reward prediction error (RPE). The cue is given a more positive value and causes no more changes from the tonic dopamine signalling in the future. Zack et al. recently published a comprehensive review on the uncertainty in gambling as a key factor for the behaviour to become an addiction [17]. In gambling there are no reliable cues for reward prediction and winning can always be seen as “better than expected”. Therefore, the idea was proposed that this uncertainty causes an extraordinary change in dopamine signalling based on constant RPEs, which leads to a pathological sensitisation of the reward system and gambling becomes addictive. This theory is supported by experimental findings, where increased dopamine release has been found to code the uncertainty in gambling [18]. Nevertheless, whether the addictive character of gambling is directly translated by changes in dopamine signalling at the NAc remains subject to controversies.

### Alterations in striatal dopamine signalling in GD

Many contradictory studies have been published on the alterations within striatal dopamine signalling in GD, often based on positron emission tomography (PET) measurements [19]. On the one hand, there is some evidence for an elevation in the dopamine transmission in connection to typical characteristics of gambling behaviour. Increased binding of dopamine to inhibitory striatal D_2_ and D_3_ receptors has been found to positively correlate with symptom severity in GD patients but not in healthy controls, for instance higher impulsivity, increased excitement and alertness, or impaired decision making in a reward context [20–23]. Furthermore, the dopamine synthesis capacity has been found to be elevated in GD patients compared to a control group [24]. Amphetamine, which decreases the dopamine reuptake into the presynapse and hence increases the signalling, has been found to prime gambling motivation and induces a higher striatal dopamine release only in patients with GD, not in healthy controls [25, 26]. Modafinil, another dopamine reuptake inhibitor, was able to raise the tendency to be drawn toward rewards in GD patients in contrast to a placebo [27]. The D_2_ antagonist haloperidol, which acts indirectly activating on dopamine signalling through inhibition blockage, can enhance the rewarding effects and priming caused by gambling within GD patients despite no observed effect in the control group [28].

On the other hand, several measurements have shown no difference between GD patients and healthy controls regarding striatal dopamine signalling. The striatal D_2_ and D_3_ receptor availability has been found to be the same in GD patients and controls [20, 29]. Additionally, the treatment of GD with the proposed D_2_ antagonist olanzapine has shown no effect in comparison to a placebo [30, 31]. Hence, the findings regarding the striatal dopamine signalling in GD are inconclusive to date. There seems to be evidence for some alterations, but replicable data is rare.

### The association between dopaminergic medication and GD

Within the last 20 years, the evidence for an association between developing GD and the intake of dopaminergic medication has grown. One of the first case reports was published in 2000, where 10 patients with Parkinson’s disease (PD) under levodopa treatment had developed GD and the authors speculated on the altered dopaminergic tone being the neurobiological explanation [32]. This theory was specified by another research group, proposing the idea that the overstimulation of the mesolimbic dopamine system could be the mechanism behind dopamine agonists causing GD [33]. More case reports were published and a dose-response relationship between dopamine agonists and impulse-control disorders was observed [34–37]. In 2010, the DOMINION study received a lot of attention, which investigated impulse-control disorders in 3090 PD patients in a cross-sectional and multicentral design in Northern America. Within all patients, 5.0% had developed GD and the risk was higher under dopamine agonist treatment (6.9% with GD) [38]. Multiple other studies were published on the subject within the last decade, suggesting a connection between dopamine agonists, respectively dopamine modulators, and the diagnosis GD [39–44].

According to previous research, certain dopaminergic medication entails a higher risk for impaired impulse control and GD development than other substances. In comparison to other dopamine agonists, especially pramipexole and ropinirole seem to correlate with increased GD rates. Both pharmaceuticals were developed as agonists for the D2-like receptor family, thus activating the inhibitory pathways of dopamine signalling, and are prescribed as part of the dopamine replacement therapy in PD but also against restless legs syndrome [45–48]. Pramipexole and ropinirole have a higher affinity for the D_3_ receptor compared with the D_2_ receptor, which has been proposed as one possible explanation for their increased property to cause addictive and impulse-control disorders [49]. Seeman et al. could show that the proportion of patients that develop an impulse-control disorder directly correlates with D_3_-selectivity over D_2_ in dopamine agonists.

Another substance, that has been associated with an increased likelihood for developing GD, is the atypical antipsychotic aripiprazole, mainly prescribed against schizophrenia and bipolar disorder [50]. The partial dopamine agonism of aripiprazole results in its stabilising effect on dopamine levels, acting as a modulator [51]. Similar to pramipexole and ropinirole, aripiprazole shows highest affinity for D_2_ and D_3_ receptors, which could explain its impact on the development of GD.

Even if the connection between dopamine agonists, respectively aripiprazole, and an impaired impulse control could not be revealed on a molecular basis yet, the association has been shown and recognised increasingly within in the last years [52–55]. Nevertheless, more evidence based on large study populations is needed to raise more attention and find solutions for the issues with certain dopaminergic medication regarding adverse psychiatric effects. Therefore, this cross-sectional study aimed to investigate the prescribed drugs within all diagnosed GD patients in the Swedish population. The association between a pramipexole /ropinirole or an aripiprazole prescription and a GD diagnosis was analysed statistically, while certain other factors that could contribute to a higher likelihood of a GD diagnosis were eliminated by choosing adequate reference groups.

## Materials and methods

### Register material

Patient data was provided by the Swedish National Board of Health and Welfare and obtained from the Swedish National Patient Registry, which is based on in-patient and specialised out-patient health care records, and the Swedish Prescribed Drug Register. The study was approved by the Swedish Ethical Review Authority under the file number 2019–01559. Since the data was analysed anonymously, consent from the participants was not obtained. For the study population, all patients in Sweden with the diagnosis pathological gambling (F63.0 according to ICD-10) between 2005 and 2019 were selected (n = 3689). Each case was paired with two age- and gender-matched controls from the total population register by Statistics Sweden with the goal to analyse the association between the exposure to the medication and the development of gambling disorder independently of the variables age and gender. This resulted in a total study population of 11 067 individuals.

The register material from the Swedish National Patient Registry included data on diagnoses according to ICD-10, age at treatment, gender, and the date of treatment. These data were combined with information from the Swedish Prescribed Drug Register containing the prescription date and the full anatomical therapeutic chemical (ATC) codes for the drug classes N02, N03, N04, N05, N06, N07B, R06AD01, R06AD02 and R06AD52.

### Statistical analysis

The aim of this study was to explore a possible association between the prescription for a certain medication and a GD diagnosis using the software IBM SPSS Statistics 26 to process and analyse the data. In preparation for the analysis, the different files received from the Swedish National Board of Health and Welfare were merged into one main file, containing all relevant information for each patient. Based on the ATC codes, binary variables were created for each medication group or pharmaceutical in question, for instance “pramipexole prescription/no pramipexole prescription” or “psychotropic drug prescription/no psychotropic drug prescription”. The same method was used to create binary variables for certain diagnoses, such as “GD/no GD” or “PD/no PD”, from the full list of diagnoses made in the in-patient and specialised out-patient health care.

In a next step, adequate subgroups within the total study population were identified. The goal was to select patients with a similar severity of disease to make individuals with a prescription for the pharmaceuticals in question as comparable as possible to the whole reference group. Since the counts of PD and restless legs syndrome patients were very low (32, respectively 27 patients in total), the subgroup for analysing dopamine agonists (pramipexole and/or ropinirole) was chosen to be all patients with any dopaminergic drug prescription (ATC code N04B, n = 180), which includes dopamine agonists but also for instance levodopa. Within this group, patients resembled each other in age, gender, and a possible PD diagnosis, not influenced by whether they had a prescription for a dopamine agonist (n = 149) or only another dopaminergic drug (n = 31) (see S1 Appendix). As a reference group for an aripiprazole prescription all patients with schizophrenia, schizotypal, delusional, and other non-mood psychotic disorders were selected (F20-29 diagnoses according to ICD-10, n = 389). Even within this group the age and gender distribution were similar in between patients with an aripiprazole prescription (n = 170) and without an aripiprazole prescription (n = 219) (see S2 Appendix) and the general mental health status could be expected to be comparable.

To determine the association between a dopamine agonist or an aripiprazole prescription with a GD diagnosis, chi-square tests were performed within the chosen subgroups. The tests were based on the binary variables “dopamine agonist prescription/no dopamine agonist prescription”, respectively “aripiprazole prescription/no aripiprazole prescription”, and “GD diagnosis/no GD diagnosis”. In both cases, all expected counts were higher than 5 per cell, making a chi-square test applicable.

Additionally, a binary logistic regression with the dependent variable “GD diagnosis/no GD diagnosis” was performed for each group to examine the effect size. Covariates were gender, age, dopamine agonist prescription, and psychotropic drug prescription, respectively gender, age, and aripiprazole prescription. The covariate psychotropic drug prescription was applied in the group with dopaminergic drug prescriptions to distinguish a compromised mental health status in general from GD as a specific psychiatric diagnosis. The variable included all prescriptions within psycholeptics, psychoanaleptics, drugs used in addictive disorders and antihistamines used as tranquillisers (ATC codes N05, N06, N07B, R06AD01, R06AD02, and R06AD52). Since the subgroup for analysing aripiprazole prescriptions was chosen according to the psychiatric diagnosis of the patients, the use of the covariate psychotropic drug prescription was assumed to be redundant in this case. The number of patients with the outcome GD diagnosis was large enough to apply four, respectively three covariates, with 116 GD patients in the group with dopaminergic prescriptions and 307 GD patients in the subgroup chosen to analyse aripiprazole prescriptions. For the chi-square tests and the logistic regressions, a significance level of 0.05 was applied and all hypothesis testing was performed two-tailed on independent samples.

Considering a possible causal relation, the time that passed in between the first dopamine agonist or aripiprazole prescription and the GD diagnosis was calculated based on the earliest date of prescription and the earliest date of the GD diagnosis for each patient. This analysis was performed on all 104 patients with a dopamine agonist prescription and a GD diagnosis, and all 151 patients with an aripiprazole prescription and a GD diagnosis.

## Results

### The association between a dopamine agonist prescription and GD in patients with any dopaminergic drug prescription

The chi-square test showed a significant association between a dopamine agonist prescription and a GD diagnosis with a P-value (P) of 0.001. Of all patients with any dopaminergic drug prescription (n = 180), including dopamine agonists but also for instance levodopa, 116 (64.4%) had been diagnosed with GD. While 104 of 149 patients (69.8%) with a dopamine agonist prescription had a GD diagnosis, only 12 out of 31 patients (38.7%) without a dopamine agonist prescription had a GD diagnosis (see Table 1).

**Table 1:** Frequency counts and row percentages for dopamine agonist prescriptions and GD diagnoses in patients with any dopaminergic prescription.

		No GD diagnosis	GD diagnosis	Total
**No dopamine agonist prescription**	Count	19	12	31
	Expected Count	11.0	20.0	31.0
	Row percentage	61.3%	38.7%	100.0%
**Dopamine agonist prescription**	Count	45	104	149
	Expected Count	53.0	96.0	149.0
	Row percentage	30.2%	69.8%	100.0%
**Total**	Count	64	116	180
	Expected Count	64.0	116.0	180.0
	Row percentage	35.6%	64.4%	100.0%

In the logistic regression, the covariates gender and age had no significant influence on the outcome GD diagnosis. The odds ratio (OR) for having a GD diagnosis was 3.2 (95% confidence interval (CI) = 1.4–7.6; P = 0.008) for patients who had received a dopamine agonist prescription compared to patients without a dopamine agonist prescription. In the same regression model, the OR for having a GD diagnosis when having a psychotropic drug prescription was 5.8 (95% CI = 1.9–17.5; P = 0.002) in comparison to patients without any psychotropic drug prescription. The complete characterisation of the applied regression model can be found in the S3 Appendix.

When the date of the first dopamine agonist prescription was compared with the date of the GD diagnosis, a majority of the patients had received the prescription first (81 of 104). The time that passed between prescription and diagnosis where 3.5 years as a median with an interquartile range (IQR) between 0.6 and 6.8 years, including all 104 patients regardless if prescription or diagnosis occurred first (see S5 Appendix).

### The association between an aripiprazole prescription and GD in F20-29 patients

Even for an aripiprazole prescription the association with a GD diagnosis could be confirmed by a chi-square test with a significance of P < 0.001. Within the 389 patients with a F20-29 diagnosis, 307 (78.9%) had a GD diagnosis. Out of 170 patients with an aripiprazole prescription a total of 151 (88.8%) had a GD diagnosis, while only 156 out of 219 patients (71.2%) without the prescription had been diagnosed with GD (see Table 2).

**Table 2:** Frequency counts and row percentages for aripiprazole prescriptions and GD diagnoses in patients with F20-29 diagnoses.

		No GD diagnosis	GD diagnosis	Total
**No aripiprazole prescription**	Count	63	156	219
	Expected Count	46.2	172.8	219.0
	Row percentage	28.8%	71.2%	100.0%
**Aripiprazole prescription**	Count	19	151	170
	Expected Count	35.8	134.2	170.0
	Row percentage	11.2%	88.8%	100.0%
**Total**	Count	82	307	389
	Expected Count	82.0	307.0	389.0
	Row percentage	21.1%	78.9%	100.0%

The covariates gender and age had no significant effect on the outcome GD diagnosis in the logistic regression performed. However, the OR for having a GD diagnosis when having an aripiprazole prescription was 3.4 (95% CI = 1.9–6.1) with a significance of P < 0.001 in comparison to patients without an aripiprazole prescription. A full description of the regression model is included in the S4 Appendix.

Most of the patients had received their first aripiprazole prescription before the GD diagnosis (101 of 151). The median time difference including all 151 patients was 1.5 years between prescription and diagnosis with an IQR between -1.3 years and 4.9 years (see S5 Appendix).

## Discussion

The results of the statistical analysis were convincing and coherent. Dopamine agonists as well as aripiprazole had an association with an increased risk for GD. Given the study design, these findings can be expected to have a high reliability and validity. The study population was large with more than 11 000 individuals, of which 3689 were diagnosed with GD. Since the data was obtained from a nationwide public institution, it can be assumed to represent the situation in Sweden accurately. Furthermore, the reference groups for the statistical analysis were selected to reach the highest comparability possible. The controls were age- and gender-matched and the overall health state was expected to be homogenous within the reference groups. Thus, a GD diagnosis is presumably linked to the intake of medication rather than a poor health condition in general. The OR for dopamine agonists was calculated while using a psychotropic drug prescription as another covariate. Both covariates showed significant ORs, and it can therefore be assumed that GD is not only associated with a compromised mental health state but also with dopamine agonist intake independently. For analysing the effects of aripiprazole, a subgroup with similar psychiatric diagnoses was chosen. Thus, GD presumably is connected to aripiprazole intake and is not only a comorbidity to other psychiatric diagnoses.

Since a GD diagnosis was treated as a binary variable, the course of disease could not be taken into consideration in this study. Future study designs should focus on the progression of the disorder under certain medication in more detail, for instance by using patient questionnaires, which could also take subclinical problematic gambling into account. Due to the cross-sectional study design, a causal relationship between the medication and GD can only be a speculation, given that most patients received their prescriptions before their diagnosis. For exploring causality, a longitudinal study would be more adequate, in which follow-up screenings for GD and impaired impulse control could be performed on patients who received the medication in question. Another limitation of this study is the missing information about the actual medication intake since the data was obtained from the prescription register. To what extent the patients made use of the prescribed medication is uncertain. Even this issue could be avoided in the future by following up on certain patients instead of using register data.

In contrast to the well-recognised DOMINION study on impulse-control disorders as a reaction to dopamine agonists [38], this study did not focus on PD patients. It was an important step to further support the results found in PD patients with findings in non-PD patients, since PD is characterised by a pathological dopamine transmission, which might even interfere with the dopaminergic reward system. The results of this study are in line with the DOMINION study, as with many other publications on the subject. While the association between increased GD rates and dopamine agonists and modulators starts to be well established raising assumptions about a causal relationship, the understanding on a molecular level is very limited so far. Only few studies have addressed the direct connection between the pharmacodynamics of dopaminergic drugs and the neurobiology of GD. Activating the striatal D_3_ receptor could be the possible mode of action for dopamine agonists, which would explain a dose-dependent relationship and pramipexole and ropinirole as agonists that increase the risk for GD the most. The pharmacodynamics of aripiprazole on the other hand, are more complex and still subject to debate [51]. It acts as a partial agonist on D2-like receptors, thus yielding an antagonistic effect under high endogenous dopamine levels and functioning as a modulator. Aripiprazole additionally interacts with serotonergic receptors and has been observed to show functional selectivity on postsynaptic D2 receptors, activating a different intracellular signalling pattern than the natural ligand dopamine [56]. Therefore, the neurochemical link between aripiprazole and developing GD is even less understood to date than the influence of dopamine agonists on the disorder.

In summary, our study puts additional emphasis on the possible effects of dopamine agonists and modulators on GD development. Even if institutions like the U.S. Food and Drug Administration (FDA) have recognised the problem, the knowledge needs to be established broadly in the primary care centres. One beneficial measure could be to assess a patient’s background and risk factors for GD and impulse-control disorders, before prescribing dopaminergic medication. If, for instance, the general mental health state indicates a higher likelihood to develop GD, it could be advisable to choose another pharmaceutical if possible, or to closely monitor the patient for side effects related to impaired impulse-control.

## Conclusions

The association between the intake of certain dopaminergic pharmaceuticals and the development of GD has started to become an international consensus over the last years. This study provides further evidence for GD being a possible response to dopamine agonists or aripiprazole. A causal relationship is likely, and the dopaminergic reward system seems to be the linkage between the medication and the addictive disorder. Since the molecular background of GD is only partly understood to date, it remains unclear how dopamine agonists or aripiprazole can influence its pathophysiology. The possible association with GD should be taken into consideration when prescribing dopamine agonists and aripiprazole, and for patients at risk for a compromised impulse control other medication seems more suitable. Furthermore, an extended knowledge on the matter is necessary to develop dopaminergic medication that can act more specifically on its intended target without causing impulse-control disorders and addictions. Revealing the molecular mechanisms behind GD would in addition contribute to a better understanding of addictive disorders in general and would open new possibilities for pharmacotherapy in this field.

## Supporting information

S1 Appendix Characterisation of the subgroup “Patients with any dopaminergic drug prescription”. (DOCX)

S2 Appendix Characterisation of the subgroup “Patients with a F20-29 diagnosis”. (DOCX)

S3 Appendix Logistic regression in the subgroup “Patients with any dopaminergic drug prescription”. (DOCX)

S4 Appendix Logistic regression in the subgroup “Patients with a F20-29 diagnosis”. (DOCX)

S5 Appendix Comparison between the date of GD diagnosis and the first prescription date for DAs and aripiprazole. (DOCX)
