# Supplementary material for: Increased risk for developing gambling disorder under the treatment with pramipexole, ropinirole, and aripiprazole: A nationwide register study in Sweden
Source: PLoS One. 2021 Jun 1;16(6):e0252516. doi: 10.1371/journal.pone.0252516 (PMC8168838; doi:10.1371/journal.pone.0252516)
Supplement: S1 Appendix — (DOCX) [file pone.0252516.s001.docx]

**S1 Appendix. Characterisation of the subgroup “Patients with any dopaminergic drug prescription”**

**Gender**

**Table S1A. Gender distribution depending on a DA prescription.**

|  |  | **Male** | **Female** | **Total** |
| --- | --- | --- | --- | --- |
| **No DA prescription** | Count | 18 | 13 | 31 |
|  | Expected Count | 18.4 | 12.6 | 31.0 |
|  | Row percentage | 58.1 % | 41.9 % | 100.0 % |
| **DA prescription** | Count | 89 | 60 | 149 |
|  | Expected Count | 88.6 | 60.4 | 149.0 |
|  | Row percentage | 59.7 % | 40.3 % | 100.0 % |
| **Total** | Count | 107 | 73 | 180 |
|  | Expected Count | 107.0 | 73.0 | 180.0 |
|  | Row percentage | 59.4 % | 40.6 % | 100.0 % |

A chi-square test showed no significant association between gender and a DA prescription (P = 0.86).

**Age**

**Figure S1A. Boxplot of the age distribution depending on a DA prescription.**


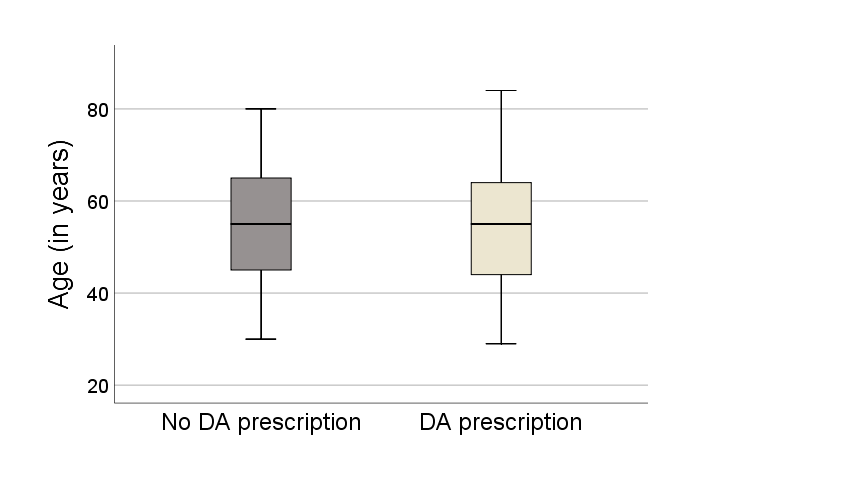


**Table S1B. Percentiles of the age distribution depending on a DA prescription.**

|  |  | **5^th^** | **10^th^** | **25^th^** | **50^th^** | **75^th^** | **90^th^** | **95^th^** |
| --- | --- | --- | --- | --- | --- | --- | --- | --- |
| **Age** (in years) | No DA prescription | 30.6 | 35.2 | 45.0 | 55.0 | 65.0 | 76.0 | 78.2 |
|  | DA prescription | 32.0 | 35.0 | 44.0 | 55.0 | 64.5 | 74.0 | 76.0 |

**PD diagnosis**

**Table S1C. PD diagnosis depending on a DA prescription.**

|  |  | **No PD diagnosis** | **PD diagnosis** | **Total** |
| --- | --- | --- | --- | --- |
| **No DA prescription** | Count | 25 | 6 | 31 |
|  | Expected Count | 26.0 | 5.0 | 31.0 |
|  | Row percentage | 80.6 % | 19.4 % | 100.0 % |
| **DA prescription** | Count | 126 | 23 | 149 |
|  | Expected Count | 125.0 | 24.0 | 149.0 |
|  | Row percentage | 84.6 % | 15.4 % | 100.0 % |
| **Total** | Count | 151 | 29 | 180 |
|  | Expected Count | 151.0 | 29.0 | 180.0 |
|  | Row percentage | 83.9 % | 16.1 % | 100.0 % |

A chi-square test showed no significant association between a PD diagnosis and a DA prescription (P = 0.59).
