# Supplementary material for: Increased risk for developing gambling disorder under the treatment with pramipexole, ropinirole, and aripiprazole: A nationwide register study in Sweden
Source: PLoS One. 2021 Jun 1;16(6):e0252516. doi: 10.1371/journal.pone.0252516 (PMC8168838; doi:10.1371/journal.pone.0252516)
Supplement: S2 Appendix — (DOCX) [file pone.0252516.s002.docx]

**S2 Appendix. Characterisation of the subgroup “Patients with a F20-29 diagnosis”**

**Gender**

**Table S2A. Gender distribution depending on an ARI prescription.**

|  |  | **Male** | **Female** | **Total** |
| --- | --- | --- | --- | --- |
| **No ARI prescription** | Count | 181 | 38 | 219 |
|  | Expected Count | 176.8 | 42.2 | 219.0 |
|  | Row percentage | 82.6 % | 17.4 % | 100.0 % |
| **ARI prescription** | Count | 133 | 37 | 170 |
|  | Expected Count | 137.2 | 32.8 | 170.0 |
|  | Row percentage | 78.2 % | 21.8 % | 100.0 % |
| **Total** | Count | 314 | 75 | 389 |
|  | Expected Count | 314.0 | 75.0 | 389.0 |
|  | Row percentage | 80.7 % | 19.3 % | 100.0 % |

A chi-square test showed no significant association between gender and an ARI prescription (P = 0.27).

**Age**

**Figure S2A. Boxplot of the age distribution depending on an ARI prescription.**


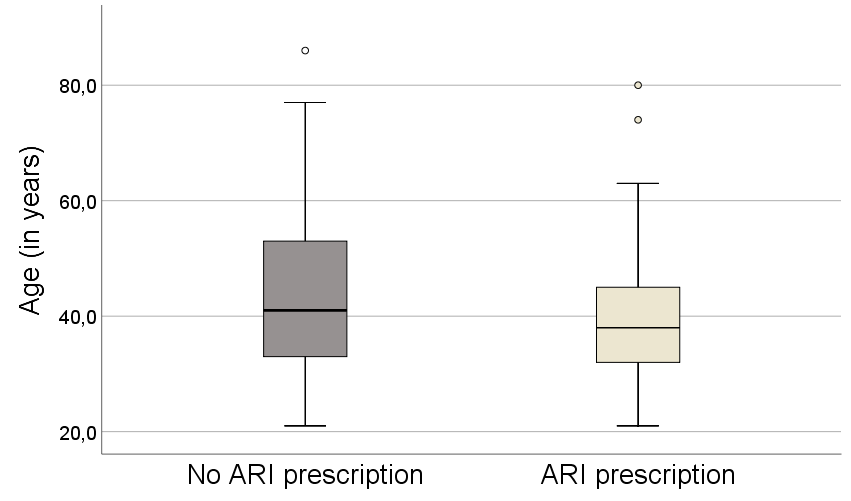


**Table S2B. Percentiles of the age distribution depending on an ARI prescription.**

|  |  | **5^th^** | **10^th^** | **25^th^** | **50^th^** | **75^th^** | **90^th^** | **95^th^** |
| --- | --- | --- | --- | --- | --- | --- | --- | --- |
| **Age** (in years) | No ARI prescription | 25.0 | 29.0 | 33.0 | 41.0 | 53.0 | 61.0 | 64.0 |
|  | ARI prescription | 26.0 | 28.0 | 32.0 | 38.0 | 45.0 | 53.9 | 56.5 |
