# Supplementary material for: Increased risk for developing gambling disorder under the treatment with pramipexole, ropinirole, and aripiprazole: A nationwide register study in Sweden
Source: PLoS One. 2021 Jun 1;16(6):e0252516. doi: 10.1371/journal.pone.0252516 (PMC8168838; doi:10.1371/journal.pone.0252516)
Supplement: S3 Appendix — (DOCX) [file pone.0252516.s003.docx]

**S3 Appendix. Logistic regression in the subgroup “Patients with any dopaminergic drug prescription”**

**Block 0: Beginning Block**

| **Classification Table^a,b^** | | | | | |
| --- | --- | --- | --- | --- | --- |
|  | **Observed** | | **Predicted** | | |
|  |  |  |  | | Percentage Correct |
|  |  |  | No gambling disorder | Gambling disorder |  |
| **Step 0** |  | No gambling disorder | 0 | 64 | .0 |
|  |  | Gambling disorder | 0 | 116 | 100.0 |
|  | Overall Percentage | |  |  | 64.4 |
| a. Constant is included in the model. | | | | | |
| b. The cut value is .500 | | | | | |

| **Variables in the Equation** | | | | | | | |
| --- | --- | --- | --- | --- | --- | --- | --- |
|  | | **B** | **S.E.** | **Wald** | **df** | **Sig.** | **Exp(B)** |
| **Step 0** | Constant | .595 | .156 | 14.587 | 1 | .000 | 1.812 |

| **Variables not in the Equation** | | | | | |
| --- | --- | --- | --- | --- | --- |
|  | | | **Score** | **df** | **Sig.** |
| **Step 0** | Variables | DA | 10.824 | 1 | .001 |
|  |  | Psychotropic drugs | 17.132 | 1 | .000 |
|  |  | Gender | .385 | 1 | .535 |
|  |  | Age | 3.016 | 1 | .082 |
|  | Overall Statistics | | 19.310 | 25.236 | 4 |

**Block 1: Method = Enter**

| **Omnibus Tests of Model Coefficients** | | | | |
| --- | --- | --- | --- | --- |
|  | | **Chi-square** | **df** | **Sig.** |
| **Step 1** | Step | 25.321 | 4 | .000 |
|  | Block | 25.321 | 4 | .000 |
|  | Model | 25.321 | 4 | .000 |

| **Model Summary** | | | |
| --- | --- | --- | --- |
| **Step** | **-2 Log likelihood** | **Cox & Snell R Square** | **Nagelkerke R Square** |
| 1 | 208.973^a^ | .131 | .180 |
| a. Estimation terminated at iteration number 4 because parameter estimates changed by less than .001. | | | |

| **Hosmer and Lemeshow Test** | | | |
| --- | --- | --- | --- |
| **Step** | **Chi-square** | **df** | **Sig.** |
| **1** | 6.196 | 8 | .625 |

| **Contingency Table for Hosmer and Lemeshow Test** | | | | | | | | | | | | |
| --- | --- | --- | --- | --- | --- | --- | --- | --- | --- | --- | --- | --- |
|  | | | **No gambling disorder** | | | | **Gambling disorder** | | | | **Total** | |
|  |  |  | Observed | | Expected | | Observed | | Expected | |  |  |
| **Step 1** | | 1 | 14 | | 14.223 | | 4 | | 3.777 | | 18 | |
|  |  | 2 | 10 | | 10.122 | | 8 | | 7.878 | | 18 | |
|  |  | 3 | 9 | | 7.127 | | 9 | | 10.873 | | 18 | |
|  |  | 4 | 8 | | 6.696 | | 13 | | 14.304 | | 21 | |
|  |  | 5 | 3 | | 4.959 | | 14 | | 12.041 | | 17 | |
|  |  | 6 | 3 | | 4.924 | | 15 | | 13.076 | | 18 | |
|  |  | 7 | 4 | | 4.141 | | 12 | | 11.859 | | 16 | |
|  |  | 8 | 6 | | 4.277 | | 12 | | 13.723 | | 18 | |
|  |  | 9 | 2 | | 3.954 | | 16 | | 14.046 | | 18 | |
|  |  | 10 | 5 | | 3.577 | | 13 | | 14.423 | | 18 | |
| **Classification Table^a^** | | | | | | | | | | | |  |
|  | **Observed** | | | | | **Predicted** | | | | | |  |
|  |  |  |  |  |  |  | | | | Percentage Correct | |  |
|  |  |  |  |  |  | No gambling disorder | | Gambling disorder | |  |  |  |
| **Step 1** |  | | | No gambling disorder | | 24 | | 40 | | 37.5 | |  |
|  |  |  |  | Gambling disorder | | 13 | | 103 | | 88.8 | |  |
|  | Overall Percentage | | | | |  | |  | | 70.6 | |  |
| a. The cut value is .500 | | | | | | | | | | | |  |

| **Variables in the equation** | | | | | | | | | | |
| --- | --- | --- | --- | --- | --- | --- | --- | --- | --- | --- |
|  | | **B** | **S.E.** | **Wald** | **df** | **Sig.** | **Exp(B)** | **95 % CI for EXP(B)** | |  |
|  |  |  |  |  |  |  |  | **Lower** | **Upper** |  |
| **Step 1^a^** | DA | 1.164 | .438 | 7.061 | 1 | .008 | 3.201 | 1.357 | 7.552 |  |
|  | Psychotropic drugs | 1.761 | .561 | 9.872 | 1 | .002 | 5.820 | 1.940 | 17.460 |  |
|  | Gender | .108 | .344 | .098 | 1 | .754 | 1.114 | .567 | 2.188 |  |
|  | Age | .017 | .012 | 1.982 | 1 | .159 | 1.017 | .993 | 1.042 |  |
|  | Constant | -2.992 | 1.018 | 8.634 | 1 | .003 | .050 |  |  |  |
| a. Variable(s) entered on step 1: DA, Psychotropic drugs, Gender, Age. | | | | | | | | | | |
