# Supplementary material for: Increased risk for developing gambling disorder under the treatment with pramipexole, ropinirole, and aripiprazole: A nationwide register study in Sweden
Source: PLoS One. 2021 Jun 1;16(6):e0252516. doi: 10.1371/journal.pone.0252516 (PMC8168838; doi:10.1371/journal.pone.0252516)
Supplement: S4 Appendix — (DOCX) [file pone.0252516.s004.docx]

**S4 Appendix. Logistic regression in the subgroup “Patients with a F20-29 diagnosis”**

**Block 0: Beginning Block**

| **Classification Table^a,b^** | | | | | |
| --- | --- | --- | --- | --- | --- |
|  | **Observed** | | **Predicted** | | |
|  |  |  |  | | Percentage Correct |
|  |  |  | No gambling disorder | Gambling disorder |  |
| **Step 0** |  | No gambling disorder | 0 | 82 | .0 |
|  |  | Gambling disorder | 0 | 307 | 100.0 |
|  | Overall Percentage | |  |  | 78.9 |
| a. Constant is included in the model. | | | | | |
| b. The cut value is .500 | | | | | |

| **Variables in the Equation** | | | | | | | |
| --- | --- | --- | --- | --- | --- | --- | --- |
|  | | **B** | **S.E.** | **Wald** | **df** | **Sig.** | **Exp(B)** |
| **Step 0** | Constant | 1.320 | .124 | 112.781 | 1 | .000 | 3.744 |

| **Variables not in the Equation** | | | | | |
| --- | --- | --- | --- | --- | --- |
|  | | | **Score** | **df** | **Sig.** |
| **Step 0** | Variables | ARI | 17.801 | 1 | .000 |
|  |  | Gender | .141 | 1 | .708 |
|  |  | Age | .051 | 1 | .821 |
|  | Overall Statistics | | 19.310 | 3 | .000 |

**Block 1: Method = Enter**

| **Omnibus Tests of Model Coefficients** | | | | |
| --- | --- | --- | --- | --- |
|  | | **Chi-square** | **df** | **Sig.** |
| **Step 1** | Step | 20.358 | 3 | .000 |
|  | Block | 20.358 | 3 | .000 |
|  | Model | 20.358 | 3 | .000 |

| **Model Summary** | | | |
| --- | --- | --- | --- |
| **Step** | **-2 Log likelihood** | **Cox & Snell R Square** | **Nagelkerke R Square** |
| 1 | 380.320^a^ | .051 | .079 |
| a. Estimation terminated at iteration number 5 because parameter estimates changed by less than .001. | | | |

| **Hosmer and Lemeshow Test** | | | |
| --- | --- | --- | --- |
| **Step** | **Chi-square** | **df** | **Sig.** |
| **1** | 7.266 | 8 | .508 |

| **Contingency Table for Hosmer and Lemeshow Test** | | | | | | |
| --- | --- | --- | --- | --- | --- | --- |
|  | | **No gambling disorder** | | **Gambling disorder** | | **Total** |
|  |  | Observed | Expected | Observed | Expected |  |
| **Step 1** | 1 | 16 | 13.012 | 23 | 25.988 | 39 |
|  | 2 | 10 | 11.454 | 27 | 25.546 | 37 |
|  | 3 | 10 | 10.997 | 27 | 26.003 | 37 |
|  | 4 | 11 | 10.955 | 28 | 28.045 | 39 |
|  | 5 | 11 | 10.235 | 29 | 29.765 | 40 |
|  | 6 | 6 | 8.215 | 34 | 31.785 | 40 |
|  | 7 | 3 | 4.866 | 36 | 34.134 | 39 |
|  | 8 | 6 | 4.326 | 32 | 33.674 | 38 |
|  | 9 | 7 | 4.035 | 31 | 33.965 | 38 |
|  | 10 | 2 | 3.905 | 40 | 38.095 | 42 |

| **Classification Table^a^** | | | | | |
| --- | --- | --- | --- | --- | --- |
|  | **Observed** | | **Predicted** | | |
|  |  |  |  | | Percentage Correct |
|  |  |  | No gambling disorder | Gambling disorder |  |
| **Step 1** |  | No gambling disorder | 0 | 82 | .0 |
|  |  | Gambling disorder | 0 | 307 | 100.0 |
|  | Overall Percentage | |  |  | 78.9 |
| a. The cut value is .500 | | | | | |

| **Variables in the equation** | | | | | | | | | | |
| --- | --- | --- | --- | --- | --- | --- | --- | --- | --- | --- |
|  | | **B** | **S.E.** | **Wald** | **df** | **Sig.** | **Exp(B)** | **95 % CI for EXP(B)** | |  |
|  |  |  |  |  |  |  |  | **Lower** | **Upper** |  |
| **Step 1^a^** | ARI | 1.230 | .291 | 17.842 | 1 | .000 | 3.421 | 1.933 | 6.053 |  |
|  | Gender | -.262 | .323 | .657 | 1 | .418 | .769 | .408 | 1.450 |  |
|  | Age | .012 | .011 | 1.154 | 1 | .283 | 1.012 | .990 | 1.034 |  |
|  | Constant | .709 | .569 | 1.553 | 1 | .213 | 2.033 |  |  |  |
| a. Variable(s) entered on step 1: ARI, Gender, Age. | | | | | | | | | | |
