# Supplementary material for: Increased risk for developing gambling disorder under the treatment with pramipexole, ropinirole, and aripiprazole: A nationwide register study in Sweden
Source: PLoS One. 2021 Jun 1;16(6):e0252516. doi: 10.1371/journal.pone.0252516 (PMC8168838; doi:10.1371/journal.pone.0252516)
Supplement: S5 Appendix — (DOCX) [file pone.0252516.s005.docx]

**S5 Appendix. Comparison between the date of GD diagnosis and the first prescription date for DAs and ARI**

**DAs** (104 patients)

**Figure S4A. Boxplot of the time between the first DA prescription and the GD diagnosis.**


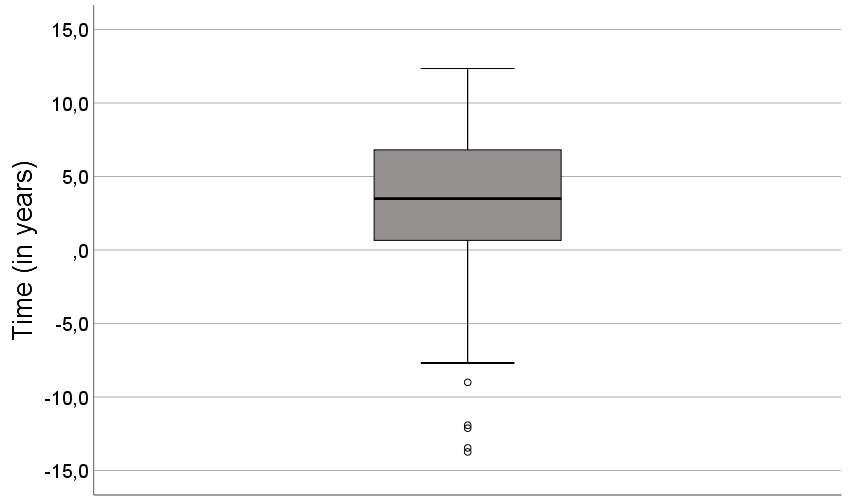


**Table S4A. Percentiles of the time between the first DA prescription and the GD diagnosis.**

|  |  | **5^th^** | **10^th^** | **25^th^** | **50^th^** | **75^th^** | **90^th^** | **95^th^** |
| --- | --- | --- | --- | --- | --- | --- | --- | --- |
| **Time** (in years) | | -8.7 | -4.3 | 0.6 | 3.5 | 6.8 | 9.4 | 11.4 |

**ARI** (151 patients)

**Figure S4B. Boxplot of the time between the first ARI prescription and the GD diagnosis.**


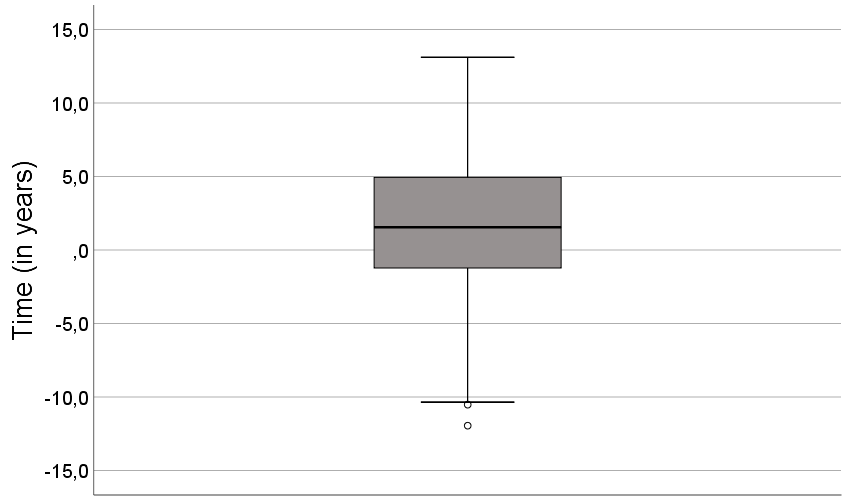


**Table S4B. Percentiles of the time between the first DA prescription and the GD diagnosis.**

|  |  | **5^th^** | **10^th^** | **25^th^** | **50^th^** | **75^th^** | **90^th^** | **95^th^** |
| --- | --- | --- | --- | --- | --- | --- | --- | --- |
| **Time** (in years) | | -7.4 | -4.8 | -1.3 | 1.5 | 4.9 | 8.4 | 10.8 |
